# The complete mitochondrial genome of *Hemisphaerius rufovarius* Walker, 1858

**DOI:** 10.1080/23802359.2020.1780967

**Published:** 2020-07-07

**Authors:** Liang-jing Yang, Lin Yang, Zhi-Min Chang, Yu-Jie Zhang, Xiang-Sheng Chen

**Affiliations:** a Institute of Entomology, Guizhou University, Guiyang, P.R. China; b The Provincial Special Key Laboratory for Development and Utilization of Insect Resources, Guizhou University, Guiyang, P.R. China; c Office of Academic Affairs, Liupanshui normal College, Liupanshui, P.R. China; d College of Animal Science, Guizhou University, Guiyang, P.R. China

**Keywords:** *Hemisphaeriusrufovarius*, Issidae, mitogenome, phylogenetic analysis

## Abstract

In this study, we have sequenced and annotated the complete mitochondrial genome (mitogenome) of *Hemisphaeriusrufovarius* (Hemiptera: Fulgoroidea: Issidae) for the first time,the mitogenome is 15,955 bp (GenBankNo. MT210096), includes13 PCGs, 2 rRNAs, 22 tRNAs and one putative control region (D-loop). The AT content of this mitogenomeis 78.3% (A 47.7%,T30.6%, C 13.3%, and G 8.4%). Most the PCGs started with ATN or TTG(nad5), and ended with TANor single T. The result ofPhylogenetic tree showed a close relationship among the families Issidae, Flatidae and Ricaniidae.

The Family Issidae 1839 is composed of 1,068 specieswith a broad distribution in word (Bourgoin 2020), is harmful for the host plant that includes woody and herbaceous plants. However, only the complete mitochondrial genome (mitogenome) of the species *Dentatissusdamnosus*had been sequenced in last ten years (Nan Song et al. 2010). *Hemisphaeriusrufovarius* (Hemiptera: Fulgoroidea: Issidae) isa ubiquitous species of Issidae in south of China, Malaysia (Borneo), Myanmar (ex Burma), Thailand, and Vietnam (Bourgoin 2020), is a serious pest of Lauraceae tree. In this study, the mitogenome sequences of *H. rufovarius*are sequenced to help to understand the phylogenic relationship of Fulgoroidea and the evolution ofIssidae. The total genome DNA extracted from the male samples which wascaptured from Mengla County, Yunnan province, China (101°21′E, 21°96′N, H 626 m) in 12 June 2019. The specimen (Accession number: GUGC2019/06/12-43 YLJ XTL 2males) and voucher specimen’s genome DNA (YLJ M 2019/9/18-10)were deposited in The Institute of Entomology, Guizhou University, Guiyang, China (GUGC).The mitogenome sequences of *H. rufovarius* were sequenced by next-generation sequencing method (illumine HiSeq X 10) (http://hannonlab.cshl.edu/fastx_toolkit/index.html), and assembled using Geneious 10.2.2 (http://www.geneious.com) (Kearse et al. 2012), annotated and conducted using MITOZ (Meng et al. 2019). The complete chloroplast genome sequence of *H. rufovarius* has been deposited in GenBank with the accession number MT210096 (The NCBI genbank accession link: https://www.ncbi.nlm.nih.gov/nuccore/MT210096).

The complete mitogenome of *H. rufovarius* is 15,955 bp in length (GenBankNo: MT210096), consists of 13 protein-coding genes (PCGs),2 ribosomal RNA genes (rRNAs) and 22 transfer RNA genes (tRNAs) and one large non-coding region (D-loop: [A + T]-rich region). D-loop is 1707 bp, exists between *12S* rRNAand *trnI*. The overall bases composition complete mitogenomeare47.7% A, 30.6% T, 13.3% C and 8.4% G, with AT content rich (78.3%).AT skew ((A − T)/(A + T)) and GC skew ((G ˗ C)/(G + C))are 0.219, −0.227, respectively. All the 13PCGs started with ATN, excepted nad5 (TTG), ended with TAN ora single T residue. AlltRNAsare presented the typical cloverleaf structures, excepted *tRNA^Ser(AGN).^* which DHU arm is not a typical neck ring structure. The mitogenome of *H. rufovarius* includes 19 intergenicspacers, totals 230 bp, ranges in size from 1 to 33 bp, the longest one located between *trnY*and *trnW*, and includes 9 gene overlaps, totals 47 bp, fluctuates in size from 1 to 13 bp. All PCGs of *H. rufovarius*is 10,708 bp totally, the shortest one and the longest one are *atp8* (158 bp) and *nad5* (1, 746 bp), respectively.

MEGA7.00 (Kumar et al. 2016) was used to construct the maximum likelihood (ML) tree based on complete mitogenome sequences of *H. rufovarius* and another 17 related species which belonged to differently family of the Fulgoroidea. The phylogenetic tree confirmed that the Fulgoroidea was a monophyly with high node values (BS ≥ 91). As the topologies tree showed, two species (*H. rufovarius* and *D. damnosus*) of Issidaewas clustered together; Issidae was placed near the middle and included in a clade with Flatidae and Ricaniidae in the following relationship (BS = 100): (Issidae + (Flatidae + Ricaniidae)), that were consistent with Xu et al. (2019) (Figure 1).

**Figure 1.**
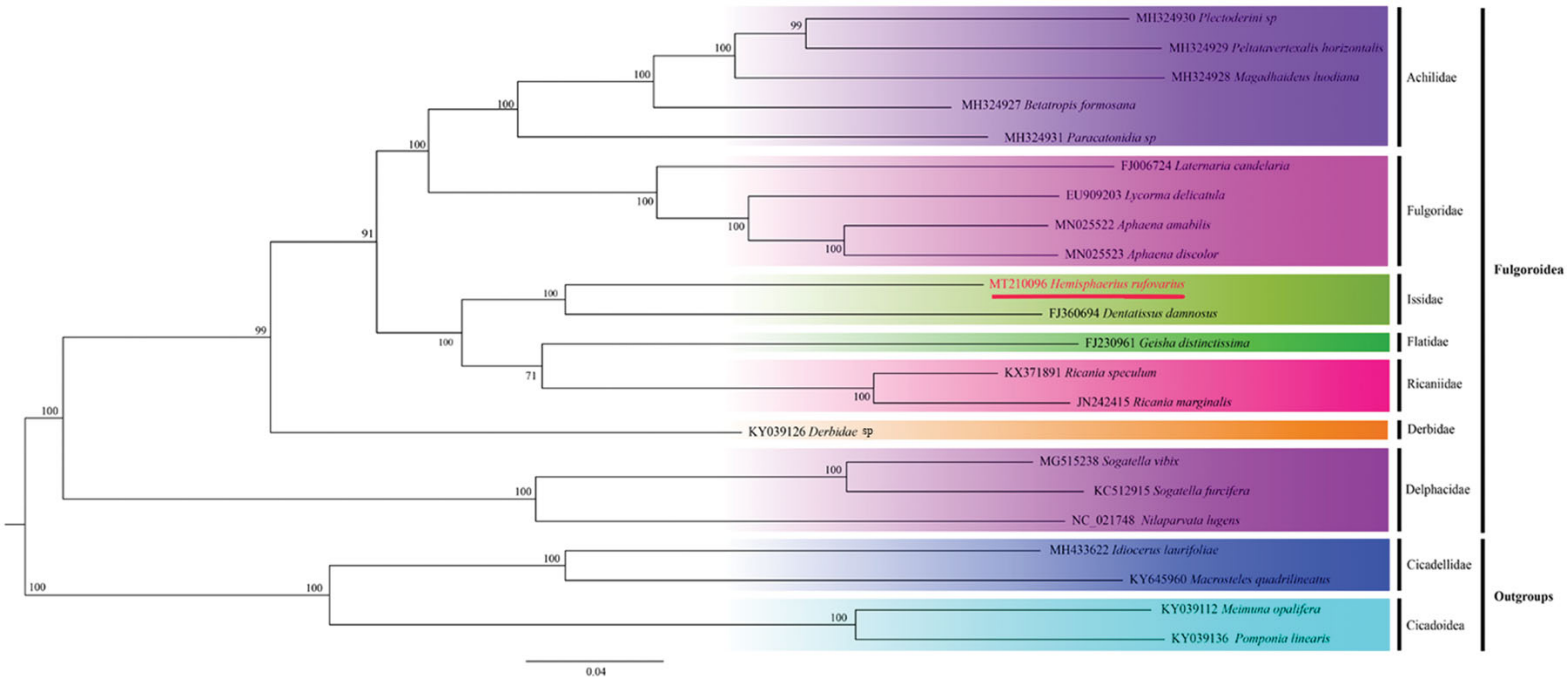
The maximum likelihood tree based on 22 the complete mitochondrial genome sequences. 18 in-group (Fulgoroidea) and 4 out-group (2 Cicadellidae and 2 Cicadoider), respectively.
